# 5-(4*H*)-Oxazolones and Their Benzamides as Potential Bioactive Small Molecules

**DOI:** 10.3390/molecules25143173

**Published:** 2020-07-11

**Authors:** Evangelos Mavridis, Eleftherios Bermperoglou, Eleni Pontiki, Dimitra Hadjipavlou-Litina

**Affiliations:** Department of Pharmaceutical Chemistry, School of Pharmacy, Faculty of Health Sciences, Aristotle University of Thessaloniki, 54124 Thessaloniki, Greece; emavridis.pharm@gmail.com (E.M.); lefterisberberoglou@gmail.com (E.B.); epontiki@pharm.auth.gr (E.P.)

**Keywords:** oxazolones, benzamides, antioxidant activities, anti-inflammatory activities, lipoxygenase inhibition, lipid peroxidation, docking studies

## Abstract

The five membered heterocyclic oxazole group plays an important role in drug discovery. Oxazolones present a wide range of biological activities. In this article the synthesis of 4-substituted-2-phenyloxazol-5(4*H*)-ones from the appropriate substituted aldehydes via an Erlenmeyer–Plochl reaction is reported. Subsequently, the corresponding benzamides were produced via a nucleophilic attack of a secondary amine on the oxazolone ring applying microwave irradiation. The compounds are obtained in good yields up to 94% and their structures were confirmed using IR, ^1^H-NMR, ^13^C-NMR and LC/MS data. The in vitro anti-lipid peroxidation activity and inhibitory activity against lipoxygenase and trypsin induced proteolysis of the novel derivatives were studied. Inhibition of carrageenin-induced paw edema (CPE) and nociception was also determined for compounds **4a** and **4c**. Oxazolones **2a** and **2c** strongly inhibit lipid peroxidation, followed by oxazolones **2b** and **2d** with an average inhibition of 86.5%. The most potent lipoxygenase inhibitor was the bisbenzamide derivative **4c**, with IC_50_ 41 μM. The benzamides **3c**, **4a**–**4e** and **5c** were strong inhibitors of proteolysis. The replacement of the thienyl moiety by a phenyl group does not favor the protection. Compound **4c** inhibited nociception higher than **4a**. The replacement of thienyl groups by phenyl ring led to reduced biological activity. Docking studies of the most potent LOX inhibitor highlight interactions through allosteric mechanism. All the potent derivatives present good oral bioavailability.

## 1. Introduction

Current novel therapeutic approaches suggest that multifunctional compounds with diverse biological properties offer significant advantages in the treatment of complicated diseases. Since inflammation is a complicated phenomenon in which several different factors are implicated, pleiotropic agents will offer additional beneficial effects. Inflammation is defined as a protective mechanism and it is the natural response of the biological system to various stimuli. It is well-established that excessive chronic inflammation is linked to reactive oxygen species, oxidative stress [1] leading to several diseases. Consequently, inflammation and oxidative environment are in a vicious circle of damaging healthy cells and increased antioxidant activity could only break into that circle.

The inflammatory process starts from arachidonic acid metabolism by three enzymatic routes: cyclooxygenase (COX), lipoxygenase (LOX) and monooxygenase cytochrome P450 (CYP) to eicosanoids [2]. The COX route leads to pro-inflammatory prostaglandins (PGs), thromboxane and other prostanoids; while the 5-, 12- and 15-LOX route leads to 5-, 12- or 15-hydro(-pero)xy-eicosatetraenoic acids [H(P)ETEs] and leukotrienes and lipoxins (LXs). Finally the cytochrome P450 monooxygenase route leads to different epoxyeicosatetraenoic acids (EETs) and diHETEs [3]. COX presents two isoforms the constitutive form (COX-1) implicated in the physiological production of prostaglandins (PGs), important for maintenance actions (e.g., stomach cytoprotection) and the inducible form (COX-2) associated with inflammation [4].

The importance of five-membered heterocyclic rings, such as isoxazoline and isoxazole is known in drug discovery [5,6,7]. Marketed antibiotics **I** such as cloxacillin, dicloxacillin, flucloxacillin and oxacillin bear the 3-arylisoxazole moiety in the side chain [8]. Recent literature mentions the importance of glucose-derived spiro-isoxazolines **II** against type 2 diabetes acting as anti-hyperglycemic agents through glycogen phosphorylase (GP) inhibition [9]. Moreover, roxifiban acetate (**III**), is a well-known antiplatelet agent (platelet glycoprotein IIb/IIIa receptor antagonist) used in percutaneous coronary intervention [10] while ISO-1 (**IV**) inhibits macrophage migration inhibitory factor (MIF), a proinflammatory cytokine [11].

**Figure 1:**
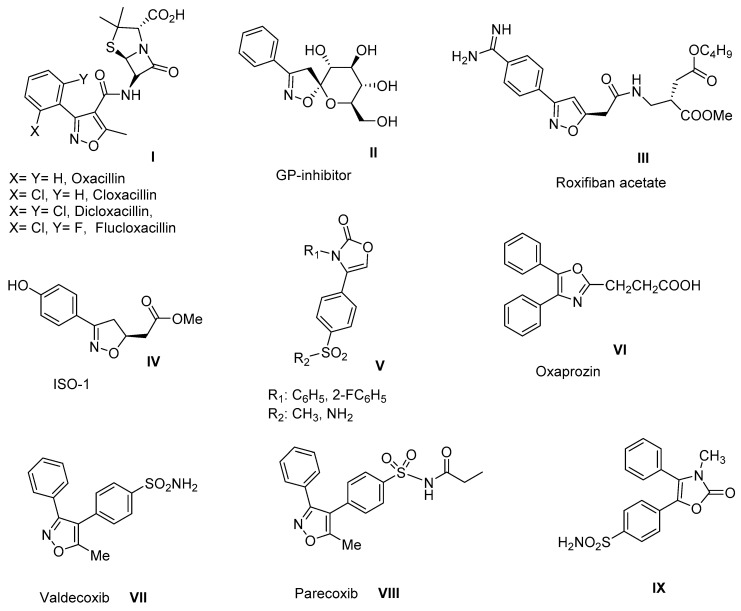
Representative biologically active oxazole-derivatives.

Furthermore, many oxazolone derivatives have been found to present potent cycloxygenase-2 (COX-2) inhibitory activity, such as the oxazolone derivative **V** bearing a sulfonamide moiety on the 4-phenyl ring, which also presented remarkable activity in vivo [12]. Additionally, the oxazole ring has been linked with the COX-inhibition e.g., oxaprozin (**VI**) which is a non-selective COX-1 and COX-2 inhibitor [13], valdecoxib (**VII**) which is a non-selective COX-2 inhibitor [14] and parecoxib (**VIII**), the sulfonamide-based prodrug of valdecoxib, the only parenterally administered coxib available to date [14,15]. In the literature a series of 4,5-diphenyl-2-oxo-3*H*-1,3-oxazole derivatives **IX** have been reported presenting selective COX-2 inhibition [16] (Figure 1).

The oxazolone group is implicated as an intermediate in the synthesis of different compounds such as amino alcohols, amides [17], amino acids [18,19] and dyes [18,20]. Oxazolones are heterocyclic compounds which can be used as versatile building blocks in organic synthesis, as they contain numerous reactive sites allowing a diversity of possible modifications. Their reactivity (nucleophilic attack to the carbon atom at position 5 of the oxazolone ring) makes them excellent substrates for the synthesis e.g., of enol acetate and benzoxazinone derivatives, phenylpyruvic acid, imidazolinones, amino acids and triazinones [21,22].

**Figure 2:**
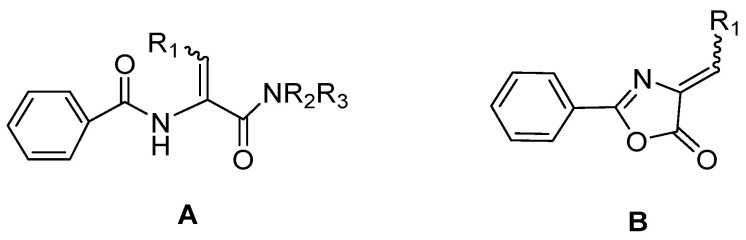
Structure of benzamides **A** and 4-substituted-2-phenyloxazol-5(4*H*)-ones **B**.

Based on the above, a number of new benzamides was designed guided by quantitative structure activity relationship (QSAR) results [23] and synthesized from appropriate substituted 5-(4*H*)-oxazolones. Benzamides **A** (Figure 2) are biologically active agents demonstrating antibacterial, antiproteolytic-antiviral, antischistosomal and anti-inflammatory activities [24,25,26,27]. On the other hand 5-(4*H*)-oxazolones **B** show a variety of biological activities: analgesic, antimicrobial, antidiabetic, anti-inflammatory as well as inhibition of trypsin and tyrosinase [28,29,30] (Figure 2).

The design principle was aimed at combining the synergistic property of biological potent ArC=C-C(=O) group to get new amides that might act as effective pleiotropic bioactive agents. Yet another objective of the study was to evaluate the effect of steric and electronic parameters on anti-inflammatory activities, to optimize the activity through systematic modification of the substituents and to take under consideration isosterism and druglikeness properties.

## 2. Results and Discussion

### 2.1. Chemistry

**Scheme 1:**
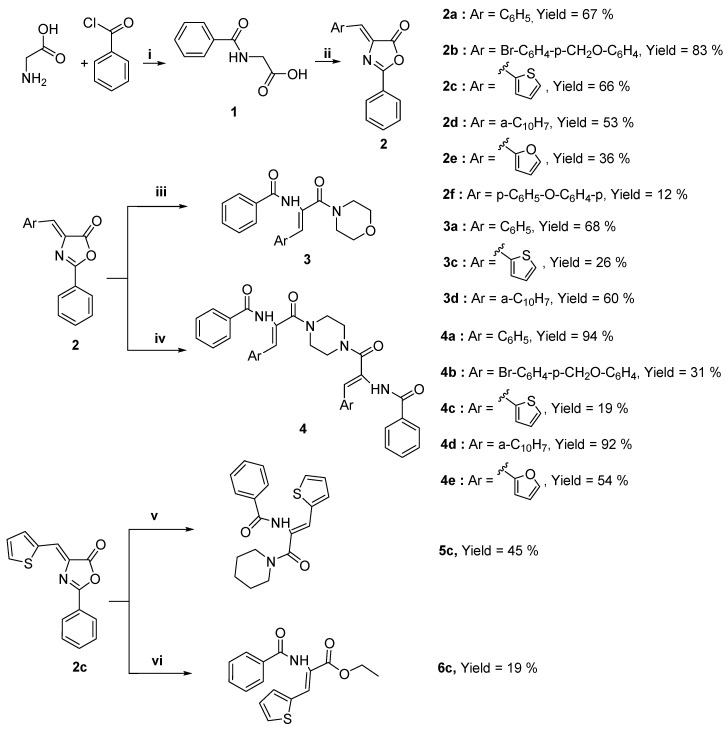
Synthetic route for target compounds **2a–2f, 3a, 3c, 3d, 4a–e, 5b** and **6b**. *Reagents and conditions*: (i) (1) 10% NaOH, 80 °C, 30 min; (2) HCl, H_2_O (ii) Method A: ArCHO, Ac_2_O, AcONa, reflux; Method B: ArCHO, Ac_2_O, AcONa, MW, 100 °C, 15 min; Method C: ArCHO, Ac_2_O, 5 mol.% I_2_, MW, 90 °C, 20 min; (iii) Method D: morpholine, toluene, reflux; Method E: morpholine, AcOEt, MW, 80 °C, 15 min; (iv) Method F: piperazine, toluene, reflux; Method G: piperazine, toluene, TEA, MW, 110 °C, 10–20 min; Method H: piperazine, EtOH, rt; Method I: piperazine, MEG, MW, 120 °C, 5 min; (v) piperidine, toluene, MW, 100 °C, 15 min; (vi) EtOH, 75 °C, 30 min.

The compounds were synthesized according to the general procedure presented in Scheme 1. The condensation of glycine with benzoyl chloride leads to compound **1**. Equimolar amounts of hippuric acid (**1**) and of the appropriate aldehyde were condensed with a stoichiometric amount of fused sodium acetate, in the presence of acetic anhydride as the dehydrating agent [21]. An Erlenmeyer–Plochl reaction was used for the preparation of 4-substituted-2-phenyloxazol-5(4*H*)-ones **2a–2f**. Subsequently, benzamides **3a**, **3c**, **3d** and **4a–4e** were produced via a nucleophilic attack of an appropriate secondary amine (morpholine and piperazine, respectively) on the oxazolone ring. It should to be noticed that under the experimental conditions, the nucleophilic attack of piperidine and ethanol on **2c** resulted in products **5c** and **6c**, respectively. For the synthesis of the compounds, known and/or modified procedures and simple techniques were applied, which led to good yields. We focused on the use of microwave irradiation in case of **2a**, **2c** and **2d**. This is an environmentally friendly and rapid method, with good results.

From the literature, itis generally accepted that in the Erlenmeyer-Plochl reaction [31] *Z* isomers are obtained. Also, the configuration of the ring opened products is the same as that of the parent oxazolone [32]. Compounds **2a**, **2c**, **2d**, **2e**, **2f**, **3a**, **6c** have been synthesized previously under different experimental conditions [33,34,35,36,37,38,39,40,41,42,43,44]. The structures of the new compounds were confirmed by IR, ^1^H-NMR, MS (ΕSI) and elemental analysis and they were found to be consistent with the proposed structures. All the oxazolones presented a characteristic double absorption in the IR (KBr) [1792–1780 cm^−1^ (C=O)] due to the Fermi resonance. Benzamides presented the expected ^1^H-NMR signals of olefinic protons [δ (ppm): 5.83–6.68] in the Z configuration of the double bond. The ^1^H-NMR value for the olefinic proton of **6c** was recorded at higher δ values (7.39 ppm). The ^13^C-NMR value for the carbonyl amide is in accordance with the referred values in similar cases. The LC-MS results point to the presence of [M + H]^+^, [M + Na]^+^, [M + K]^+^ and [2M + Na]^+^.

### 2.2. In Silico Determination of Lipophilicity as Clog P 

Lipophilicity is an important physicochemical property in relation to the biological activity and biodistribution. We theoretically calculated the lipophilicity values of all compounds as clog *P* using the CLOGP Program of Biobyte Corp. [45].

### 2.3. Biological Evaluation

Reactive oxygen species (ROS) are continuously produced as byproducts of cell metabolism. Depending on their nature, some are highly toxic and rapidly detoxified by various cellular enzymatic and non-enzymatic mechanisms. Their engagement in pathological processes is due to their extreme reactivity and their tendency to initiate and participate in chain reactions. Detoxification of reactive oxygen species is paramount to the survival of all aerobic life forms. Normally, a number of defense mechanisms have been involved by organisms against these highly reactive species by using enzymes and naturally occurring antioxidants. Antioxidants in general, even at low concentration, significantly delay or prevent oxidation of easily oxidizable substrates.

Azo compounds, like AAPH, generating free radicals through spontaneous thermal decomposition are useful for in vitro studies of free radical production. Production of conjugated diene hydroperoxide by oxidation of sodium linoleate in an aqueous solution is monitored at 234 nm. This assay can be used to follow oxidative changes and to understand the contribution of each tested compound. Oxazolones **2a** and **2c** strongly inhibit lipid peroxidation, followed by oxazolones **2b** and **2d** (Table 1). The average inhibition of these four compounds is 86.5%, demonstrating that the Ar-substituent (bulky or not) doesn’t significantly affect the result. It seems that the presence of moiety Ar (Scheme 1) is mainly correlated with the antioxidant result. The nature of the heteroatom O/S in Ar substituent in the 5-membered oxazolone ring (compounds **2c** and **2e**) influences the biological response. The replacement of the thienyl group in **2c** by a furyl group (**2e**) leads to a 31% decrease of the antioxidant ability. It must be noticed that isosteric replacement of Ar = phenyl by a thienyl (**2c**, 91%) or a napthyl group (**2d**, 81%) does not induce any dramatic change. 

Among the group of morpholinylbenzamides **3c** and **3d** present higher inhibitory activity, whereas compound **3a** with Ar = phenyl exhibits 69% inhibition. Lipophilicity does not seem to play a role. The stereochemistry of the molecules might be more important. Considering the piperazinyl group of bisbenzamides, all the analogues present high anti-lipid peroxidation activity ranging from 82–99%, with **4b**, **4c**, **4d** in the 90–99% range. The replacement of the Ar substituent (phenyl by a furyl group as in **4a**, **4e**) does not influence the antioxidant ability. It seems that the Ar substituent does not offer/contribute to the overall response. On the contrary the nature of the alicyclic ring does seem to influence the anti-lipid peroxidation ability. Thus, the morpholinylbenzamide **3c** is more potent (93%) compared to the piperidinyl (**5c**, 72%). The piperazinyl bisbenzamides **4a–4e** showed high protection against lipid peroxidation.

It is well known that free radicals play an important role in inflammatory action [46]. In fact, many inhibitors of LOX have been reported to act either as radical scavengers or as inhibitors of free radical production, since lipoxygenation occurs via a carbon centered radical [47]. LOXs involvement in membrane lipid peroxidation by forming hydroperoxides in the lipid bilayer, leads their inhibitors to be considered as potential agents for the treatment of inflammatory diseases. Consequently, LOX inhibitors with antioxidant properties could be expected to offer protection in inflammation and lead to potentially effective drugs. Thus, we found interesting to test our compounds for their ability to inhibit plant LOX activity, since it has been shown that its inhibition by NSAIDs is qualitatively similar to their inhibition of the rat mast cell LOX and may be used as a simple qualitative screen for such activity [48,49].

In our experiments the compounds showed either no (**2a**, **2b**, **2c**) or low (**2d**, **2e**, **3a**, **3c**, **3d**, **5c**, **6c**) biological activity (<50%, Table 1). Among the tested compounds the most interesting representatives were the piperazinyl bis-benzamides **4b–4e** for which we were able to determine r IC_50_ values under our experimental conditions. Therefore, it must be stressed that the presence of a piperazine, by forming bisbenzamides, is crucial for the majority of our compounds in order to exert satisfactory biological activity. Compounds **4b–4e** highly inhibit LOX as well as lipid peroxidation. These results strengthen the assumption that many inhibitors of LOX may act either as radical scavengers or as inhibitors of free radical production. However, compounds **2a** and **2c** with high antioxidant activity did not present any LOX inhibition.

The synthesized derivatives have been tested for their antityrosinase activity [50]. Tyrosinase is an enzyme with a dinuclear copper centre widely distributed in Nature. Tyrosinase plays a critical role in the biosynthesis of melanin in melanocytes, considering to be the key enzyme in coloring the skin, hair, eyes [51]. Thus, inhibition of tyrosinase will offer to skin whitening effects and dipegmentation after sunburn [52]. Oxazolones **2a**–**2e** as well as the tested morpholinyl benzamides **3a**, **3c**, **3d** were not found to present any significant activity. Judging the activity of piperazinyl bisbenzamides **4a**, **4c**, **4d** it seems that they are more potent with a range from 28–42 % (**4c** > **4a** > **4d**). Again, **4c** was found to be the most active compared to the corresponding oxazolone **2c** and benzamide **3c**. However, they are less potent than kojic acid which has been used as a reference compound. This compound is the most potent tyrosinase inhibitor till now. Thus, there is a need to search for new inhibitors.

Therefore, we tested our compounds for their ability to inhibit tyrosinase in combination to LOX inhibition in order to have possible treatment in cases of hyperpigmentation in people suffering from psoriasis, etc. 

It is well known that serine proteases are associated with inflammatory responses, especially trypsin which has been implicated in host defense reactions and tissue damage [28,29,30]. Trypsin is a prime example of a serine protease that contains a catalytic triad consisting of histidine, aspartate and serine units. These three amino acids form a charge relay that serves to make the active site serine nucleophilic by modifying its electrostatic environment. The aspartate residue located in the catalytic pocket of trypsin is responsible for attracting and stabilizing positively charged lysine and/or arginine, and is, thus, responsible for the specificity of the enzyme. This means that trypsin predominantly cleaves, through ‘’activated’’ serine, proteins at the C-terminal side of the amino acids lysine and arginine except when either is bound to a C-terminal proline [28]. Inhibition of enzymatic proteolysis is of great importance because proteolysis, apart from inflammation, plays a crucial role in the premature activation of proteases themselves, resulting in the self-digestion of the pancreas, as well as in the life cycle of herpes viruses [29]. Therefore, potential antiprotease activity of our compounds could lead to multifunctional drugs.

Bisbenzamides **4a–4e** present high activity with minor differences among them. Amides of morpholine **3c** and piperidine **5c** seem to be almost equipotent. However the difference in terms of the acyclic amine did not lead to any change on the biological response. The small fluctuation in the rates concerning the inhibitory ability of the above compounds (8–9.1 μM) denotes that their individual structural characteristics slightly influence their biological action. The benzamide **6c** is the only ester and presents the highest IC_50_ value (IC_50_ = 1 μM), compared to the reference compound salicylic acid (IC_50_ = 100 μM). More studies have to be done in order to clarify the structural characteristics that determine the wide range of IC_50_ values (60–7μM for **2b**–**2e** and 33% for **2a**) (Table 1).

For the estimation of anti-inflammatory potential [30] of the tested compounds the rat carrageenin induced paw edema assay was employed as a model for acute inflammation. Indomethacin, as a reference drug, and our compounds were administered intraperitoneally in order to ensure systemic response via fast bioavailability, while carrageenin’s intradermal administration, as inflammatory agent, aimed to local response. The development of the edema induced by carrageenin has been described as a biphasic event. The early phase of the inflammatory response, lasting 0.5–1.5 h, is chiefly mediated by histamine and serotonin release inducing hyperalgesia, swelling and redness, while the later phase, lasting 3–6 h, is sustained presumably by prostaglandin release. This model reliably predicts the anti-inflammatory efficacy of the NSAIDs during the second phase, since non-steroidal anti-inflammatory drugs (NSAIDs) present weak activity in the first phase. Therefore, using this protocol we could evaluate our compounds as potential anti-inflammatory agents, as a result of inhibition of prostaglandin amplification.

**Figure 3:**
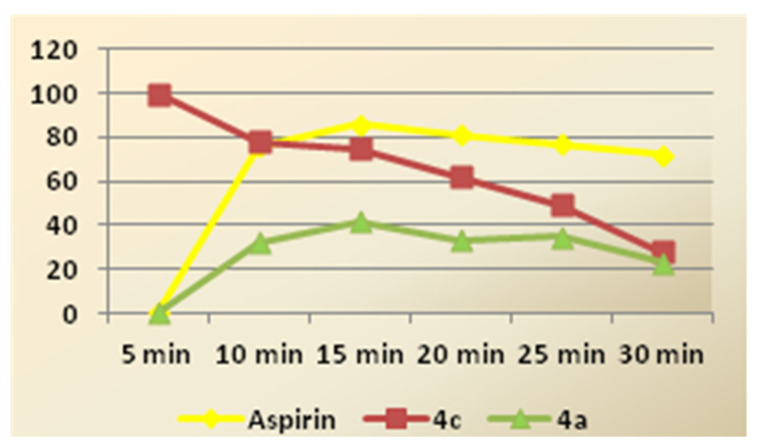
% Anti-nociception measured every 5 min for **4a**, **4c** and aspirin.

Compound **4c** was chosen to be examined because it showed generally promising antioxidative activity, anti-lipid peroxidation and LOX inhibition, while **4a** was selected in order to delineate the influence of the nature of the ring on the anti-inflammatory activity. Compound **4c** and the reference drug indomethacin seem to be equipotent, while compound **4a** presents lower decrease at an equivalent dose (Table 2). Thus, it is obvious that the replacement of thienyl by phenyl group does not favor the protection against the CPE. Compounds **4a** and **4c** were tested for their peripheral nociception using the writhing test (Figure 3). Despite the fact that acetic acid induced writhing test lacks of specialization, it remains a remarkable method for assessing peripheral analgesic activity because of its quickness, simplicity and reproducibility (Table 2).

Derivative **4c** inhibited writhing responses more effectively than **4a**. Thus the replacement of the thienyl group by the phenyl ring led to reduced biological activity. Obviously these results are in accordance with the compounds’ anti-inflammatory activity. According to Table 2, compound **4c** exhibited protective action from the very first moment, after the administration of acetic acid, compared to the reference drug aspirin whereas compound **4a** later, after the 15 min. These findings point to a different pharmacokinetic behavior however more experiments are in progress to delineate these results.

### 2.4. Molecular Properties Prediction—Lipinski “Rule of Five”

Pharmacokinetic properties should be considered in the design and synthesis of new bioactive compounds. Nowadays the traditional experimental techniques have been replaced by the use of computational tools. The predicted properties have been popular since they are taken faster and without cost [53].

Herein, we obtained and entered in the online Molinspiration software version 2018.10 (www.molinspiration.com) [54] the chemical structures and SMILES notations of the more potent representative compounds **4a**, **4b**, **4c**, **4d**, **4e**, **5c** and **6c** to calculate various molecular properties e.g., partition coefficient (log *P*), topological polar surface area (TPSA), hydrogen bond donors and acceptors, rotatable bonds, number of atoms, molecular weight and to define Lipinski’s rule of five violations, in an attempt to evaluate their drug-likeness [55].

**Figure 4:**
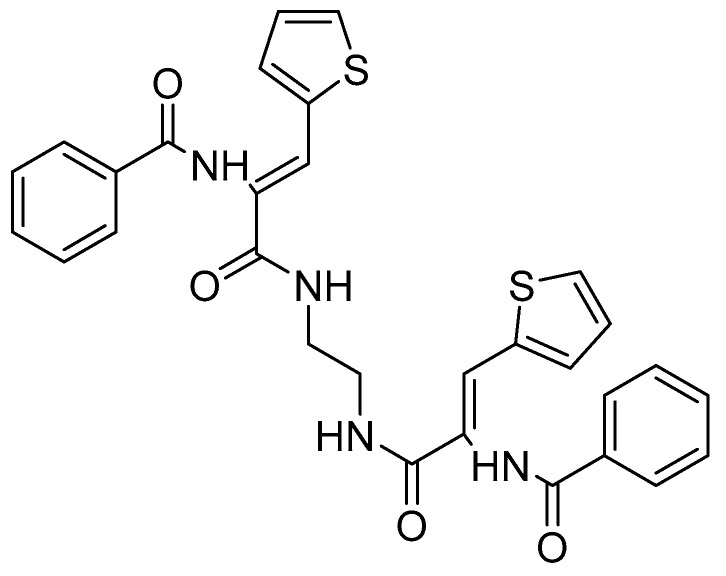
Suggested modifications—Compound **7**.

Following the Lipinski’s rule of five the presence of more than five H-bond donors, 10-H-bond acceptors, molecular weight (MW) greater than 500 and the calculated log *P* values (milogP) higher than 5, leads to poor absorption or permeation. It is a rule of thumb to define if a chemical structure with a certain bioactivity presents drug-likeness. Thus, these properties would support its behavior in humans as an active *per os* drug. Compounds **4a**, **b**, **d** with log *P* values > 5 violate the rule of 5. They might present permeability problems. However, compounds **4c**, **e** and **5c**, **6c** have lipophilicity lower than five suggesting satisfactory permeability across cell membranes. On the contrary **4c** has high MW as well as compound **4a**. Both present molecular weight higher than 500. Thus, these molecules could not be transported, diffused and absorbed. The compounds of group 4 are bisbenzamides of piperazine. Following the rule of Lipinski, replacement of the alicyclic ring by an aliphatic amine e.g., ethylenediamine (-NHCH_2_CH_2_NH-) should result in a better metabolic profile for the compounds of this group (Figure 4). Considering this modification compound **7** would be subjected to a possible proteolysis/amidolysis in vivo leading to several products. Prediction of bioactivity of compound **7** by Molinspiration [54] presents an interesting antiprotease score.

The number of hydrogen bond acceptors and donors in compounds **4a**–**e**, **5c** and **6c** obey Lipinski’s rules. Topological polar surface area (TPSA) is referred as a significant indicator of bioavailability of a molecule with certain biological activity. TPSA is correlated with the hydrogen bonding properties. Compounds **4a**, **b**, **c**, **d**, **5c** and **6c** exhibit TPSA values well below the limit of 160 Å^2^, supporting the presence of good bioavailability *per os*. The upper limit of TPSA is 90 Å^2^. The calculated results for the **4a**, **b**, **c**, **d**, **e** show that these structures are not able to cross the Blood Brain Barrier (TPSA value should be 40 Å^2^). On the contrary compounds **5c** and **6c** are able due to their lower (less than 60 Å^2^) values (Table 3).

### 2.5. Computational Studies—Docking Simulation Soybean Lipoxygenase

#### Molecular Modeling of the Synthesized Derivatives in Soybean LOX

**Figure 5:**
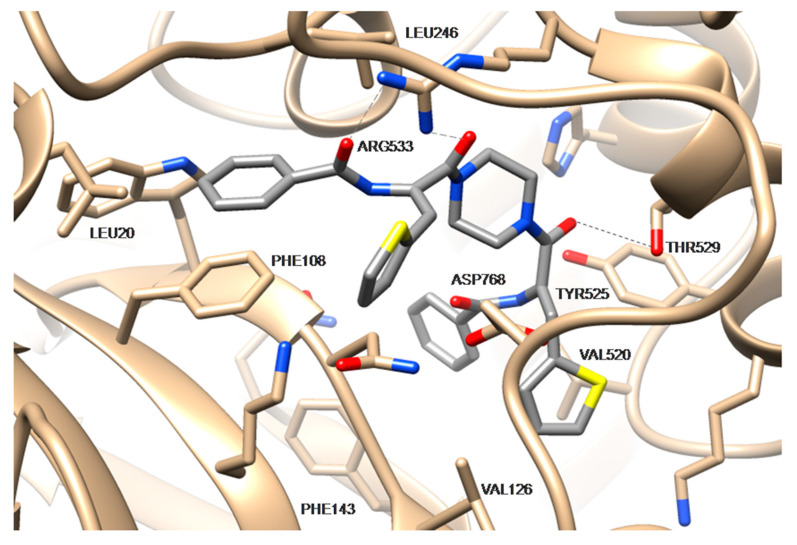
Preferred docking pose of **4c** (depicted in grey) bound to soybean lipoxygenase (LOX-1).

The synthesized derivatives have been subjected to in silico docking in accordance with the biological results. The favored docking position for the most promising bisbenzamide **4c** is shown in Figure 5. Compound **4c** has an AutoDockVina score of ‒10.6 kcal/mol binding to soybean LOX (PDB code: 3PZW). It is well known that the results from the in vitro inhibition of soybean lipoxygenase represent experimental values while docking scores are based on algorithms and scoring function calculations so a 1 to 1 correlation is difficult to achieve. Docking describes the ligand binds to the protein. It seems that the novel compounds interact with the SLOX through allosteric interactions. Compound **4c** presents weak hydrogen bonds with ARG533 and THR529 with the two carbonyl groups of the 1*H*-indene-1,3(2*H*)-dione template and hydrophobic ones with the rest of the molecule with aminoacids LEU20, PHE108, VAL126, PHE143, LEU246, VAL520, TYR525 and ASP768. Since most LOX inhibitors act as antioxidants or by scavenging free radicals [56] and the oxidation of the enzyme occurs via a carbon-centered radical on a lipid chain, it is possible that compound **4c** extends into the hydrophobic domain by blocking the substrates to the binding site and thus preventing oxidation.

## 3. Experimental Section

### 3.1. Materials and Instruments

All chemicals, solvents, chemical and biochemical reagents were of analytical grade and purchased from commercial sources (Merck KGaA, Darmstadt, Germany, Fluka Sigma-Aldrich Laborchemikalien GmbH, Hannover, Germany, Alfa Aesar, Karlsruhe, Germany and Sigma-Aldrich St. Louis, MO, USA) and used without further purification. Soybean lipoxygenase, albumin, trypsin, sodium linoleate, sodium linoleate, 2,2-azinobis-2-methyl-propanimidamine HCl (AAPH), mushroom tyrosinase were obtained from Sigma Chemical, Co. (St. Louis, MO, USA), nordihydroguairetic acid (NDGA), salycilic acid, kojic acid were purchased from the Aldrich Chemical Co. (Milwaukee, WI, USA).

Melting Points (uncorrected) were determined on a MEL-Temp II (Lab. Devices, Holliston, MA, USA). For the in vitro tests, UV-Vis spectra were obtained on a Perkin-Elmer 554 double beam spectrophotometer. Infrared spectra (film as Nujol mulls or KBr pellets) were recorded with a Perkin-Elmer 597 spectrophotometer (Perkin-Elmer Corporation Ltd., Lane Beaconsfield, Bucks, England). For the microwave irradiated reactions a 700 W CEM Microwave Discover System was used (Matthews, North Carolina, MC USA).

The ^1^H Nucleic Magnetic Resonance (NMR) spectra were recorded at 300 MHz on an AM-300 spectrometer (Bruker Analytische Messtechnik GmbH, Rheinstetten, Germany) in CDCl_3_ or DMSO using tetramethylsilane as an internal standard unless otherwise stated. ^13^C-NMR spectra were obtained at 75.5 MHz on a Bruker AM-300 spectrometer in CDCl_3_ or DMSO solutions with tetramethylsilane as internal reference unless otherwise stated. Chemical shifts are expressed in _ (ppm) and coupling constants *J* in Hz. Mass spectra were determined on a LC-MS 2010 EV system (Shimadzu, Kyoto, Japan) using MeOH as solvent. Elemental analyses for C and H gave values acceptably close to the theoretical values (±0.4%) in a Perkin-Elmer 240B CHN analyzer. Reactions were monitored by thin layer chromatography on 5554 F254 Silica gel/TLC cards (Merck and Fluka Chemie GmbH Buchs, Steinheim, Switzerland). 

### 3.2. Chemistry General Procedure

#### 3.2.1. Synthesis of *N*-Benzoylglycine Hippuric Acid (**1**)

Glycine (20 mmol) was dissolved in 20 mL of 10% NaOH and the mixture was stirred to ambient temperature for 20 min. Afterward, 20 mmol of benzoyl chloride was added in drops into the flask and the temperature of the reaction solution was raised to 80 °C for 30 min. Crushed ice was added to the solution and concentrated HCl was added dropwise until the mixture was acidified (pH 2–3). The white formed precipitate was collected by filtration and washed out with water (negative reaction for chloride). The spectral data was in agreement with literature data [33,34,35,36,37,38,39,40,41,42,43,44]. 

#### 3.2.2. Synthesis of (*Z*)-4-Arylidene-2-phenyloxazol-5(4*H*)-ones **2a–f**

Method A: To a mixture of N-benzoylglycine (**1**) (1 mmol) and fused sodium acetate (1 mmol), acetic anhydride (3 mmol) and the appropriate aldehyde (1 mmol) was added. The reaction mixture was heated until melting, and then refluxed for 2 h. The reaction process was monitored by TLC. The solution was poured into ice cold ethanol and then allowed to cool overnight. The formed precipitate was collected by filtration, washed with ice cold ethanol and boiling water, successively, and then recrystallized, if necessary, from aqueous ethanol or from water/aqueous ethanol mixture.

Method B: *N*-Benzoylglycine (**1**) (1 mmol), fused sodium acetate (1 mmol) and an appropriate aldehyde (1 mmol) were mixed in acetic anhydride (3 mmol) contained in a 10 mL microwave vial sealed by Teflon-lined rubber cap. The reaction mixture was irradiated by microwave at 300 W and 100 °C for 15 min, poured into ice cold ethanol and allowed to cool overnight, giving a precipitate which was filtered, washed with ice cold ethanol and boiling water to afford *(Z)*-4-arylidene-2-phenyloxazol-5(4*H*)-one derivatives **2a**, **2c**, **2d** (Table 4).

Method C: A mixture of *N*-benzoylglycine (1 mmol), an appropriate aldehyde (1 mmol), and molecular iodine (5 mol.%) instead of sodium acetate, in acetic anhydride (4 mmol) was irradiated by microwave at 300 W and 90 °C for 20 min. The mixture was cooled to room temperature, ice cold ethanol was added and the crude oxazolone was separated. The product was filtered and washed with ice cold ethanol/boiling water to afford the pure product **2c** (Table 4).

*(Z)-4-Benzylidene-2-phenyloxazol-5(4H)-one* (**2a**) [35,36,37,38,41]. Yield: 67%; R_f_ = 0.8 (CH_3_COOC_2_H_5–_ petroleum ether, 6:1); m.p. 165–167 °C; IR (KBr) (cm^−1^): 3066–3054 (arom. C-H), 1793, 1769 (C=O), 1652 (C=N), 1596 (C=C), 1553, 1449 (arom. C-C), 1295, 1230 (C-O); ^1^H-NMR (CDCl_3_), δ (ppm): 7.21 (s, 1H, olefinic), 7.44–7.50 (m, 3H), 7.53 (t, 2H), 7.62 (t, 1H), 8.17–8.25 (m, 4H); ^13^C-NMR (CDCl_3_), δ:126.5 (2C), 128.6 (2C), 128.7 (2C), 128.9, 129.0 (2C), 129.4, 131.2, 131.6, 131.7, 132.0, 164.7, 165.1;C_16_H_11_NO_2_ [M + H]^+^ =250. The spectral data were in agreement with the literature data.

*(Z)-4-(4-((4-Bromobenzyl)oxy)benzylidene)-2-phenyloxazol-5(4H)-one* (**2b**). Yield: 83%; R_f_ = 0.8 (CH_3_COOC_2_H_5–_petroleum ether, 6:1); m.p. 199–200 °C; IR (KBr) (cm^−1^): 3064–3054 (arom. C-H), 2919 (olefinic C-H), 1780, 1762 (C=O), 1655 (C=N), 1592 (C=C), 1509 (arom. C-C), 1298, 1252 (C-O); ^1^H-NMR (CDCl_3_), δ (ppm): 5.07–5.12 (m, 2H, aliphatic), 6.98–7.09 (m, 2H, arom.), 7.21 (s, 1H, olefinic), 7.31 (d, 2H, *J* = 9 Hz, arom.), 7.49–7.56 (m, 4H, arom.), 7.59 (t, 1H, arom.), 8.03–8.21 (m, 4H, arom.); ^13^C-NMR (CDCl_3_), δ: 69.5, 115.5 (2C), 120.8, 127.6 (2C), 128.3(2C), 129.0 (2C), 129.1, 131.0 (2C), 131.2, 131.4, 131.6, 132.0, 133.1(2C), 134.6, 161.1, 164.7, 165.2; Anal. C, H, N. (C_23_H_16_BrNO_3_) Calcd %: C: 63.61, H: 3.71, N: 3.25; Found %: C: 63.59, H: 3.91, N: 3.41; [M]^+^ = 433, [M + H]^+^ = 434 (436), [M + Na]^+^ = 456.

*(Z)-2-Phenyl-4-(thiophen-2-ylmethylene)oxazol-5(4H)-one* (**2c**) [36,41]. Yield: 66%; R_f_ = 0.7 (CH_3_COOC_2_H_5–_petroleum ether, 6:1); m.p. 179–181 °C; IR (KBr) (cm^−1^): 3072–3060 (arom. C-H), 2.916 (olefinic C-H), 1791, 1769 (C=O), 1646 (C=N), 1598 (C=C), 1552, 1414 (arom. C-C), 1295.4, 1240.8 (C-O); ^1^H-NMR (CDCl_3_), δ (ppm): 7.15–7.19 (m, 1H, thienyl), 7.49 (s, 1H, olefinic), 7.51–7.56 (m, 2H, arom.), 7.57–7.61 (m, 1H, arom.), 7.63–7.66 (m, 1H, thienyl), 7.72 (d, 1H, *J* = 6 Hz, thienyl), 8.17 (d, 2H, *J* = 6 Hz, arom.); ^13^C-NMR (CDCl_3_), δ: 123.6, 123.7, 126.2, 126.8, 127.8, 132.1, 133.0, 134.2, 134.6, 136.2, 144.4, 161.0, 168.5, 178.0; (C_14_H_9_NO_2_S) [M + H]^+^ = 256, [M + H + HCOOH]^+^ = 302. The spectral data were in agreement with the literature data.

*(Z)-4-(Naphthalen-1-ylmethylene)-2-phenyloxazol-5(4H)-one* (**2d**) [34,39,40]. Yield: 53%; R_f_ = 0.7 (CH_3_COOC_2_H_5–_petroleum ether, 6:1); m.p. 169–171 °C; IR (KBr) (cm^−1^): 3058.8–3030.8 (arom. C-H), 1793, 1779 (C=O), 1637 (C=N), 1561 (C=C), 1297, 1245 (C-O); ^1^H-NMR (CDCl_3_), δ (ppm): 7.56–7.61 (m, 3H), 7.62–7.67 (m, 3H), 7.93 (d, 1H, *J* = 6 Hz), 7.99 (d, 1H, *J* = 6 Hz), 8.16 (s, 1H, olefinic), 8.21–8.26 (m, 2H), 8.34 (d, 1H, *J* = 6 Hz), 9.04 (d, 1H, *J* = 6 Hz); ^13^C-NMR (CDCl_3_), δ: 122.7, 125.5, 126.1 (3C), 126.5, 126.8, 127.2, 128.3 (2C), 128.8, 129.4, 131.1, 131.6 (2C), 131.7, 133.2, 137.9, 159.8, 164.2; (C_20_H_13_NO_2_) [M + H]^+^ =300, [M + Na]^+^ = 322, [M + K]^+^ = 338, [M + CH_3_OH + Na]^+^ = 354. The spectra data were in agreement with the literature data.

*(Z)-4-(Furan-2-ylmethylene)-2-phenyloxazol-5(4H)-one* (**2e**) [36,41]. Yield: 36%; R_f_ = 0.6 (CH_3_COOC_2_H_5–_petroleum ether, 6:1); m.p. 165–168 °C; IR (KBr) (cm^−1^): 3109–3064 (arom. C-H), 1789 (C=O), 1648 (C=N), 1557 (C=C), 1491, 1454 (arom. C-C), 1296.3, 1231.1 (C-O); ^1^H-NMR (CDCl_3_), δ (ppm): 6.62–6.69 (m, 1H, furyl), 7.20 (s, 1H, olefinic), 7.22–7.28 (m, 1H, furyl), 7.51–7.57 (m, 2H, arom.), 7.58–7.61 (m, 1H, arom.), 7.62–7.71 (m, 1H, furyl), 8.10–8.20 (m, 2H, arom.); ^13^C-NMR (CDCl_3_), δ: 113.7, 118.2, 120.0 (2C), 128.3 (2C), 128.9, 131.3, 131.5, 133.1, 142.4, 146.5, 162.5, 165.0; C_14_H_9_NO_3_ [M + H]^+^ = 240, [M + 2CH_3_OH + H]^+^ = 304. The spectra data were in agreement with the literature data.

*(Z)-4-(4-Phenoxybenzylidene)-2-phenyloxazol-5(4H)-one* (**2f**) [43,44]. Yield: 12%; R_f_ = 0.9 (CH_3_COOC_2_H_5–_petroleum ether, 3:7); m.p. 169 °C; IR (Nujol) (cm^−1^) 3100 (arom. C-H), 1784 (C=O), 1654 (C=N); ^1^H-NMR (CDCl_3_), δ (ppm): 7.11–7.24 (m, 4H arom. and 1H olefinic), 7.39–7.53 (m, 5H), 7.58–7.63 (m, 1H), 7.70 (d, 1H, *J* = 9 Hz), 8.02–8.07 (m, 3H); ^13^C-NMR (CDCl_3_)δ: 113.0, 119.9, 120.7, 121.4, 123.9, 124.2, 126.1, 127.3, 128.5, 128.9, 130.0, 130.9, 131.3 (2C), 133.5, 135.3, 136.4, 143.3, 160.0, 165.0, 169.9, 178.0; Anal. C, H, N. Calcd %: (C_22_H_15_NO_3_) C: 77.41, H: 4.38, N: 4.06; Found %: C: 77.63, H: 4.38, N: 4.10. The spectral data were in agreement with the literature data.

#### 3.2.3. Synthesis of (*Z*)-Morpholino-benzamides **3a**, **3c**, **3d**

Method D: An equimolar quantity of arylideneoxazolone **2c** and morpholine in dry toluene were refluxed for about 2 h. The reaction process was monitored by TLC. Approximately 2/3 of the solvent was removed under reduced temperature and the remaining mixture was allowed to cool overnight to obtain solid precipitate. The precipitated solid was washed with 0.5N HCl to get the pure product **3c** (Table 4).

Method E: An equimolar quantity of arylideneoxazolones **2a**, **2d** and morpholine in ethyl acetate (2 mL) were irradiated by microwave at 50 W and 80 °C for 15 min. The solution was evaporated to dryness and washed with 0.5N HCl affording a residue that was recrystallized from aqueous ethanol/water mixture.

*(Z)-N-(3-morpholino-3-oxo-1-phenylprop-1-en-2-yl)benzamide* (**3a**) [35]. Yield: 68%; R_f_ = 0.5 (CH_3_COOC_2_H_5–_petroleum ether, 3:1); m.p. 135–138 °C; IR (Nujol) (cm^−1^): 3201 (N-H), 1657 (NH-C=O), 1646 (C=O), 1601 (C=C); ^1^H-NMR (CDCl_3_), δ (ppm): 3.76–3.85 (m, 8H, morpholino), 6.16 (s, 1H, olefinic), 7.29–7.34 (m, 1H), 7.38–7.46 (m, 6H), 7.49–7.54 (m, 1H), 7.77–7.82 (m, 2H), 8.05 (s, 1H, N-H); ^13^C-NMR (CDCl_3_), δ: 47.70 (2C), 65.7, 66.5, 119.7, 127.4, 128.3 (2C), 128.5 (2C), 128.6 (2C), 128.7 (2C), 129.1, 132.1, 134.0, 134.5, 166.1, 166.6; (C_20_H_20_N_2_O_3_) [M − H]^+^ = 335, [M + Na]^+^ = 359, [M + CH_3_OH + Na]^+^ = 391, [2M + Na]^+^ = 695. The spectral data were in agreement with the literature data.

*(Z)-N-(3-morpholino-3-oxo-1-(thiophen-2-yl)prop-1-en-2-yl)benzamide* (**3c**). Yield: 26%; R_f_ = 0.6 (CH_3_COOC_2_H_5–_petroleum ether, 3:1); m.p. 184–186 °C; IR (Nujol) (cm^−1^): 3157 (N-H), 1662 (NH-C=O), 1639 (C=O), 1608 (C=C); ^1^H-NMR (CDCl_3_), δ (ppm): 3.67–3.84 (m, 8H, morpholino), 6.43 (s, 1H, olefinic), 7.02–7.11 (m, 1H, thienyl), 7.12–7.19 (m, 1H, thienyl), 7.34–7.40 (m, 1H, thienyl), 7.41–7.57 (m, 3H, arom.), 7.87–8.02 (m, 2H, arom.), 8.12 (s, 1H, N-H); ^13^C-NMR (CDCl_3_), δ: 47.5 (2C), 66.6 (2C), 115.4, 124.3, 127.6 (2C), 128.8 (2C), 129.0 (2C), 129.1, 132.3, 133.0, 136.4, 165.7, 168.6; Anal. C, H, N. (C_18_H_18_N_2_O_3_S) Calcd % C: 63.14, H: 5.30, N: 8.18; Found %: C: 63.10, H: 5.57, N: 8.47; [M − H]^+^ = 341, [M + Na]^+^ = 365, [M + K]^+^ = 381, [2M + Na]^+^ = 707.

*(Z)-N-(3-morpholino-1-(naphthalen-1-yl)-3-oxoprop-1-en-2-yl)benzamide* (**3d**). Yield: 60%; R_f_ = 0.5 (CH_3_COOC_2_H_5–_petroleum ether, 3:1); m.p. 135–137 °C; IR (Nujol) (cm^−1^): 3265 (N-H), 1649 (NH-C=O),1634 (C=O), 1607 (C=C); ^1^H-NMR (CDCl_3_), δ (ppm): 3.75–3.93 (m, 8H, morpholino), 6.62–6.63 (m, 1H, olefinic), 7.33 (t, 2H), 7.45 (t, 1H), 7.50 (t, 1H), 7.53–7.57 (m, 2H), 7.59–7.64 (m, 3H), 7.86–7.92 (m, 2H), 7.96–7.97 (m, 1H), 8.09 (m, 1H, N-H); ^13^C-NMR (CDCl_3_), δ: 47.8 (2C), 66.7 (2C), 118.7, 125.4, 125.7, 126.2, 126.7, 126.9, 127.5, 127.8, 128.4 (4C), 129.0, 131.7, 131.9, 132.6, 133.1,134.8, 166.2, 166.8;Anal. C, H, N. (C_24_H_22_N_2_O_3_) Calcd %: C: 74.59, H: 5.75, N: 7.25; Found %: C: 74.39, H: 5.87, N: 7.03; [M − H]^+^ = 385, [M + Na]^+^ = 409, [M + CH_3_OH + Na]^+^ = 441, [2M + Na]^+^ = 795.

#### 3.2.4. Synthesis of (*Z*,*Z*′)-Piperazine-bisbenzamides **4a**–**4e**

Method F: Arylidene oxazolones **2a**–**2e** and half the stoichiometric amount of piperazine were refluxed in dry toluene. The reaction progress was monitored by TLC. Approximately 2/3 of the solvent was removed under reduced pressure and the remaining mixture was allowed to cool overnight to obtain a solid precipitate. The precipitated solid was washed with 0.5N HCl to give the pure products **4a**–**4e** (Table 4).

Method G: Arylidene oxazolones **2a**, **2c**, **2d** (2 mmol) and piperazine (1 mmol) were mixed in dry toluene (5 mL) with a catalytic amount of triethylamine, and irradiated by microwaves at 300 W, 110 °C for 10–20 min (Table 4). The mixture was allowed to cool overnight, and then the crude bisbenzamide thus separated was filtered, washed with 0.5N HCl and recrystallized, if necessary, from DMF/aqueous ethanol mixture. 

Method H: A solution of arylidene oxazolone **2a** (2 mmol) and piperazine (1 mmol) in EtOH (5 mL) was stirred at room temperature for 74 h. The ethanol was evaporated under reduced pressure and the residue was triturated with diethyl ether to give the pure product **4a**.

Method I: In a 10 mL microwave vial arylidene oxazolones **2c**, **2d** (2 mmol) with piperazine (1 mmol), in ethylene glycol (MEG, 2 mL) were mixed and then capped. The mixture was irradiated by microwaves at 300 W and 120 °C for 5 min. The contents were cooled to room temperature and then poured into cold water, filtered to give crude products, which were further purified by recrystallization from aqueous ethanol.

*N,N′-((1Z,1′Z)-piperazine-1,4-diylbis(3-oxo-1-phenylprop-1-ene-3,2-diyl))bisbenzamide* (**4a**). Yield: 94%; R_f_ = 0.3(CH_3_COOC_2_H_5–_petroleum ether, 2:1); m.p. 265–268 °C; IR (Nujol) (cm^−1^): 3204 (N-H), 1644 (NH-**C=O**), 1629 (C=O), 1606 (C=C); ^1^H-NMR (CDCl_3_), δ (ppm): 3.81–4.18 (m, 8H, piperazine), 6.20 (s, 2H, olefinic), 7.30–7.35 (m, 2H), 7.36–7.48 (m, 12H), 7.50–7.54 (m, 2H), 7.74–7.85 (m, 4H), 8.19 (s, 2H, N-H); ^13^C-NMR (CDCl_3_), δ: 43.8(4C), 126.1, 126.4, 126.5 (2C), 126.6 (2C), 127.0 (4C), 130.1 (8C), 132.3 (4C), 131.8 (2C), 132.9 (2C), 133.1 (2C), 163.4 (2C), 164.0 (2C); Anal. C, H, N. (C_36_H_32_N_4_O_4_): Calcd % C: 73.95, H: 5.52, N: 9.58; Found %: C: 73.74, H: 5.24, N: 9.31; [M − H]^+^ = 583, [M + H]^+^ = 585, [M + Na]^+^ = 607, [M + K]^+^ = 623.

*N,N′-((1Z,1′Z)-piperazine-1,4-diylbis(1-(4-((4-bromobenzyl)oxy)phenyl)-3-oxoprop-1-ene-3,2-diyl))-bis-benzamide* (**4b**). Yield: 31%; R_f_ = 0.7 (CH_3_COOC_2_H_5–_petroleum ether, 2:1); m.p. 285–286 °C; IR (Nujol) (cm^−1^): 3208 (N-H), 1668 (NH-**C=O**), 1622 (C=O), 1609 (C=C); ^1^H-NMR (DMSO), δ (ppm): 3.14–3.17 (m, 8H, piperazine), 5.02–5.10 (m, 4H), 6.44–6.58 (m, 2H, olefinic), 6.87–6.94 (m, 4H), 7.11–7.18 (m, 4H), 7.29–7.34 (m, 4H), 7.36–7.41 (m, 4H), 7.43–7.49 (m, 6H), 7.80–7.91 (m, 4H), 8.12 (s, 1H, N-H), 10.11–10.25 (m, 1H, N-H); ^13^C-NMR (DMSO), δ: 47.8 (4C), 70.8 (2C), 115.7 (4C), 120.9(2C), 121.9 (2C), 124.7 (2C), 126.8 (2C), 128.5 (8C), 129.4 (4C), 130.7 (4C), 131.9 (2C), 132.0 (4C), 134.3 (2C), 134.5 (2C), 158.6 (2C), 166.2 (2C), 166.7 (2C);Anal. C, H, N. (C_50_H_42_Br_2_N_4_O_6_): Calcd % C: 62.90, H: 4.43, N: 5.87; Found %: C: 62.87, H: 4.56, N: 5.73; [M − H]^+^ = 953, [M + Na]^+^ = 977, [M + Κ]^+^ = 993.

*N,N′-((1Z,1′Z)-piperazine-1,4-diylbis(3-oxo-1-(thiophen-2-yl)prop-1-ene-3,2-diyl))bisbenzamide* (**4c**). Yield: 19%; R_f_ = 0.3 (CH_3_COOC_2_H_5–_petroleum ether, 2:1); m.p. 292–294 °C; IR (Nujol) (cm^−1^): 3188 (N-H), 1658 (NH-C=O),1638 (C=O), 1606 (C=C); ^1^H-NMR (CDCl_3_), δ (ppm): 3.79–4.10 (m, 8H, piperazine), 6.49 (s, 2H, olefinic), 7.08–7.11 (m, 2H, thienyl), 7.14–7.17 (m, 2H, thienyl), 7.38–7.42 (m, 2H, thienyl), 7.45–7.52 (m, 4H, arom.), 7.53–7.60 (m, 2H, arom.), 7.86–7.99 (m, 4H, arom.), 8.22 (s, 2H, N-H); ^13^C-NMR (CDCl_3_), δ: 47.6 (4C), 124.2 (2C), 126.3 (2C), 128.4 (2C), 128.5 (8C), 128.6 (2C),129.2 (2C), 132.0 (2C), 134.4 (2C), 139.0 (2C); 165.8 (2C), 166.2 (2C);Anal. C, H, N. (C_32_H_28_N_4_O_4_S_2_): Calcd % C: 64.41, H: 4.73, N: 9.39; Found %: C: 64.90, H: 4.71, N: 9.02; [M − H]^+^ = 595, [M + H]^+^ = 597, [M + Na]^+^ = 619, [M + Κ]^+^ = 635.

*N,N′-((1Z,1′Z)-piperazine-1,4-diylbis(1-(naphthalen-1-yl)-3-oxoprop-1-ene-3,2-diyl))bisbenzamide* (**4d**). Yield: 92%; R_f_ = 0.2 (CH_3_COOC_2_H_5–_petroleum ether, 2:1); m.p. 282–284 °C; IR (Nujol) (cm^−1^): 3210 (N-H), 1659 (broad, C=O), 1609(C=C), 1519(H-N-C=O); ^1^H-NMR (CDCl_3_), δ (ppm): 4.00–4.22 (m, 8H, piperazine), 6.68 (s, 2H, olefinic), 7.32–7.38 (m, 4H), 7.43–7.47 (m, 2H), 7.48–7.52 (m, 2H), 7.54–7.58 (m, 4H), 7.59–7.65 (m, 6H), 7.84–7.93 (m, 6H), 7.96–8.02 (m, 2H, N-H); Anal. C, H, N. (C_44_H_36_N_4_O_4_) Calcd %: C: 77.17, H: 5.30, N: 8.18; ^13^C-NMR (CDCl_3_), δ: 47.6 (4C), 118.8 (2C); 125.4 (2C), 125.6 (2C), 126.2 (2C), 126.8 (2C), 126.9 (2C), 127.4 (2C), 127.5 (2C), 128.5 (8C), 129.0 (2C), 131.8 (2C), 131.9 (2C), 132.6 (2C), 133.1 (2C), 134.4 (2C), 166.2 (2C), 166.7 (2C);Found %: C: 77.25, H: 5.15, N: 8.28; [M − H]^+^ = 683, [M + Na]^+^ = 707, [M + Κ]^+^ = 723.

*N,N′-((1Z,1′Z)-piperazine-1,4-diylbis(1-(furan-2-yl)-3-oxoprop-1-ene-3,2-diyl))bisbenzamide* (**4e**). Yield: 54%; R_f_ = 0.5 (CH_3_COOC_2_H_5–_petroleum ether, 2:1); m.p. 293–295 °C; IR (Nujol) (cm^−1^): 3435 (N-H), 1682 (NH-**C=O**), 1623 (C=O), 1561 (C=C); ^1^H-NMR (CDCl_3_), δ (ppm): 3.80–3.95 (m, 8H, piperazine), 5.83 (s, 2H, olefinic), 6.36–6.44 (m, 2H, furyl), 6.45–6.52 (m, 2H, furyl), 7.46–7.64 (m, 8H, arom. and furyl), 7.86–7.97 (m, 4H, arom.), 9.31–9.47 (m, 2H, N-H); ^13^C-NMR (CDCl_3_), δ: 41.4 (2C), 47.0 (2C), 103.1 (2C), 111.4 (2C), 114.6 (2C), 127.4 (2C), 128.2 (8C), 132.3 (2C), 133.0 (2C), 142.7 (2C), 150.8 (2C), 164.3 (2C), 166.4 (2C); Anal. C, H, N. (C_32_H_28_N_4_O_6_): Calcd % C: 68.07, H: 5.00, N: 9.92; Found %: C: 68.26, H: 5.07, N: 10.01; [M − H]^+^ = 563, [M + Na]^+^ = 587, [M + K]^+^ = 603.

#### 3.2.5. Synthesis of (*Z*)-*N*-(3-Oxo-3-(piperidin-1-yl)-1-(thiophen-2-yl)prop-1-en-2-yl)benzamide (**5c**)

Arylideneoxazolone **2c** (1 mmol) and piperidine (1 mmol) were mixed in dry toluene (5 mL) and irradiated by microwaves at 300 W, 100 °C for 15 min (Table 4). The mixture was allowed to cool overnight, and then the solvent was removed under reduced pressure. The crude product was recrystallized from aqueous ethanol to give pure product **5c**. Yield: 45%; R_f_ = 0.7 (CH_3_COOC_2_H_5–_petroleum ether, 3:1); m.p. 181–182 °C; IR (Nujol) (cm^−1^): 3148 (N-H), 1657 (NH-**C=O**), 1636 (C=O), 1599 (C=C); ^1^H-NMR (CDCl_3_), δ (ppm): 1.65–1.73 (m, 6H, piperidine), 3.64–3.73 (m, 4H, piperidine), 6.43 (s, 1H, olefinic), 7.06–7.10 (m, 1H, thienyl), 7.11–7.16 (m, 1H, thienyl), 7.37 (d, 1H, *J* = 6 Hz, thienyl), 7.43–7.50 (m, 2H, arom.), 7.54 (t, 1H, arom.), 7.94 (d, 2H, *J* = 6 Hz, arom.), 8.09 (s, 1H, N-H); ^13^C-NMR (CDCl_3_), δ: 24.7, 25.6 (2C), 47.2 (2C), 124.4, 126.9, 127.5, 128.7 (4C), 129.0,132.1, 133.1, 136.6, 140.1, 165.4, 174.7; Anal. C, H, N. (C_19_H_20_N_2_O_2_S): Calcd % C: 67.03, H: 5.92, N: 8.25; Found %: C: 67.13, H: 5.78, N: 8.35; [M + H]^+^ = 341, [M + Na]^+^ = 363, [M + K]^+^ = 379, [M + Na + MeOH]^+^ = 395, [2M + Na]^+^ = 703.

#### 3.2.6. Synthesis of (*Z*)-Ethyl 2-benzamido-3-(thiophen-2-yl)acrylate (**6c**)

Arylideneoxazolone **2c** was heated in ethanol at 75 °C for 30 min, until pure compound **6c** was produced. Yield: 19%; R_f_ = 0.2 (CH_3_COOC_2_H_5–_petroleum ether, 1:3); m.p. 156–159 °C; IR (KBr) (cm^−1^): 3279 (N-H), 1706 (C=O ester),1649 (NH-**C=O**), 1579 (C=C), 1513 (H-N-C=O); ^1^H-NMR (CDCl_3_), δ (ppm): 1.33 (t, 3H, ester), 4.30 (q, 2H, *J* = 6 Hz, ester), 7.07 (dd, 1H, *J*_1_ = 3 Hz, *J*_2_ = 3 Hz, thienyl), 7.34 (d, 1H, *J*_1_ = 3Hz, thienyl), 7.39 (s, 1H, olefinic), 7.44 (d, 1H, *J*_2_ = 3Hz, thienyl), 7.47–7.54 (m, 2H, arom.), 7.55–7.63 (m, 1H, arom.), 7.86 (s, 1H, N-H), 7.96 (d, 2H, *J* = 9Hz, arom.); ^13^C-NMR (CDCl_3_), δ: 14.5, 61.0, 124.1, 128.3, 128.4 (4C), 128.5, 129.2, 131.3, 132.0, 134.5, 139.2, 166.3, 167.8; Anal. C, H, N. (C_16_H_15_NO_3_S): Calcd % C: 63.77, H: 5.02, N: 4.65; Found %: C: 63.45, H: 5.15, N: 4.51; [M − H]^+^ = 300, [M + H]^+^ = 302, [M +N a]^+^ = 324, [M + K]^+^ = 340, [M + CH_3_OH + Na]^+^ = 356, [2M + Na]^+^ = 625. The spectral data were in agreement with the literature data [33,42].

### 3.3. Biological Assays

#### 3.3.1. Biological In Vitro Assays

Each in vitro experiment was performed at least in triplicate and the standard deviation of absorbance was less than 10% of the mean. For the in vitro assays, a stock solution (1% DMSO in the appropriate buffer with the tested compound diluted under sonification) was prepared from which several dilutions were made with the appropriate buffer.

##### Inhibition of Linoleic Acid Lipid Peroxidation

The AAPH-induced oxidation of linoleic acid measures the ability of an antioxidant to prevent oxidation of linoleic acid sodium salt induced by alkylperoxyl radicals, generated from the water soluble azo compound 2,2′-azobis(2-amidinopropane) dihydrochloride (AAPH). The assay was performed according to our previous publications [56,57,58,59]. Trolox was used as reference compound.

##### Soybean Lipoxygenase Inhibition Study 

DMSO solution of the tested compound (stock solutions 10 mM) was incubated with sodium linoleate (0.1 mM) and 0.2 mL of soybean lipoxygenase solution (1/9 × 10^−4^ *w*/*v* in saline) at room temperature. The test follows our previous published methods [56,58,59]. The conversion of sodium linoleate to 13-hydroperoxylinoleic acid was recorded at 234 nm and compared with the standard inhibitor NDGA. Several concentrations were used to determine the IC_50_.

##### Tyrosinase Inhibition Assay 

Ten µL of compound was dissolved in DMSO (final concentration 100 µM) and incubated with 40 µL mushroom tyrosinase (5370 U/mg–1 mg/2.6 mL buffer) and 200 µL of L-Dopa (1.5 mM) in 0.1 M Na-phosphate buffer (pH = 6.5) for 20 min at 37 °C. Then the enzyme reaction was monitored by measuring the change in absorbance at 490 nm due to the formation of the DOPA-chrome and compared with the standard inhibitor kojic acid [60].

##### Inhibition of Trypsin Induced Proteolysis 

The tested compounds (10 µL) were dissolved in DMSO (final concentration 100 µM), with phosphate buffer (pH = 7.6) to give 0.005–0.1 mM solutions, and 100 µL trypsin (1 mg/10 mL) were preincubated at 37 °C for 30 min. Then, 200 µL of albumin (6 mg in 3 mL of distilled water) was added and incubated again at 37 °C for 24 h. After the incubation 1 mL trichloroacetic acid (5% *w*/*w*) was added to terminate the reaction. The mixture is centrifugated, the water layer is collected and monitored at 280 nm [61]. 

#### 3.3.2. Biological In Vivo Assays

The animals (Fisher 344 rats), which have been bred in our laboratory, were housed under standard conditions and received a diet of commercial food pellets and water ad libitum during the maintenance but they were entirely fasted during the experiment period. Both sexes were used while females pregnant were excluded. Each group was composed of 6–15 animals. Our studies were in accordance with recognized guidelines on animal experimentation (guidelines for the care and use of laboratory animals published by the Greek Government 160/1991, based on EU regulations 86/609). Rats were kept in the Centre of the School of Veterinary Medicine (EL54 BIO42), Aristotle University of Thessaloniki, which is registered by the official state veterinary authorities (presidential degree 56/2013, in harmonization with the European Directive 2010/63/EEC). The experimental protocols were approved by the Animal Ethics Committee of the Prefecture of Central Macedonia (no. 270079/2500).

##### Inhibition of the Carrageenin-Induced Edema

Edema was induced in the right hind paw of the rats by the intradermal injection of 0.1 mL 2% carrageenin in water we followed our previous published method [59]. Each group was composed of five animals. The tested compounds were suspended in water (0.0057 mmol/kg body weight), with few drops of Tween 80, ground in a mortar before use and were given intraperitoneally simultaneously. The rats were euthanized 3.5 h after carrageenin injection. The change in paw weight was compared with that in control animals (treated with water) and expressed as a percent inhibition of the edema CPE % values. Indomethacin 0.0057 mmol/kg body weight was used as reference compound. Values CPE % are the mean from two different experiments with a standard error of the mean less than 10 %.

##### Anti-Nociception—Writhing Test 

In order to study the analgesic activity of the tested compound, pain is provoked with acetic acid. Three groups for each compound were used with five animals per group. We followed our previous presented method [62,63]. After treatment with the tested compounds (0.0057 mmol/kg body weight; intraperitoneally) in group 1, 2nd group was used as a positive control (aspirin; 0.0057 mmol/kg body weight i.p.) and another group (3rd) served as the control in which saline was administered (negative control). After 30 min, 0.6% acetic acid (1 mL/kg body weight) was injected i.p. The number of writhes was recorded for each animal every 5 min during a subsequent 30 min period. 

### 3.4. Molecular Properties Prediction-Lipinski “Rule of Five”

Compounds were subjected to molecular properties prediction, drug-likeness by Molinspiration [54] (Table 3).

### 3.5. Computational Methods. Molecular Docking Studies on Soybean Lipoxygenase

UCSF Chimera was used for the visualization of the protein (PDB code: 3PZW) [64]. Water molecules were removed, missing residues were added with Modeller [65], hydrogen atoms and AMBER99SB-ILDN charges were added, and the charge on iron was set to +2.0, with no restraint applied to the iron atom and the ligands. OpenBabel was used to generate and minimize ligand 3D coordinates using the MMFF94 force field [66]. Ligand topologies and parameters were generated by AnteChamberPYthon Parser interfacE (ACPYPE) [67] using Antechamber [68]. Energy minimizations were carried out using the AMBER 99SB-ILDN force field [69] with GROMACS 4.6. Docking was performed with AutoDockVina (1.1.2) [70] applying a grid box of size 100 Å, 70 Å, 70 Å in X, Y, Z dimensions. The generation of docking input files and the analysis of the docking results was accomplished by UCSF-Chimera. Docking was carried out with an exhaustiveness value of 10 and a maximum output of 20 docking modes. 

## 4. Conclusions

The designed and synthesized benzamides derivatives of oxazolones present multi-target activity against different targets. They exhibit anti lipid peroxidation activity, inhibition of lipoxygenase and trypsin in vitro as well as anti-inflammatory and analgesic activities in vivo. In Table 5 the most significant derivatives in terms of activities are given. 

The oxazolones **2a**–**2e** as well as all the benzamides highly inhibit lipid peroxidation. In oxazolones the Ar-substituent doesn’t significantly affect the results while in bisbenzamides the nature of the alicyclic ring seems to influence the anti-lipid peroxidation ability. Among the morpholinylbenzamides, compounds **3c** and **3d** present higher inhibitory activity. 

As for the lipoxygenase inhibition the most interesting representatives were found to be the piperazinyl bisbenzamides**4b**–**4e**, with compound **4c** being the most active.

The piperazinyl bisbenzamides **4a**–**4e** exhibited inhibition of tyrosinase, with **4c** being the stronger inhibitor.

Compound **6c** was found to be an exceptional potent trypsin inhibitor and it could be used as a lead scaffold for antiprotease activity. The benzamides **3c**, **4a**–**4e** and **5c** presented strong antiprotease activity. 

Compounds **4c** and **4a**, with the best activity in the above biological assays, were selected to be tested as anti-inflammatory agents using the carrageenin-induced rat paw edema method. Compound **4c** seems to be equipotent in vivo to the reference drug indomethacin, while compound **4a** at the same dose presents lower decrease in the carrageenin paw edema and thus lower anti-inflammatory activity. Compound **4c** inhibited writhing better than **4a**.

Compound **4c** present the better multi-target abilities in vitro and in vivo and it can be considered as a lead compound with a multifunctional profile. The pharmacokinetic prediction analysis led to useful observations for the design and optimization of the ADME properties of the new derivatives. All the potent derivatives (**4a**–**e**, **5c**, **6c**) present good oral bioavailability. The bis-benzamides are not able to cross the blood brain barrier, while **5c** and **6c** display this ability. Theoretical structural modifications are in progress to delineate the role of the piperazine ring in the bisbenzamides. All the compounds have been subjected to docking studies showing that soybean LOX oxidation was prevented by blocking into the hydrophobic domain the substrates to the active site with hydrogen bonds and hydrophobic interactions.

## Figures and Tables

**Table 1:** Theoretically calculated clog *P* values; Anti-lipid peroxidation (AAPH); In vitro lipoxygenase (LOX) inhibitory activity (IC_50_ (μM) or % (100 µM)); In vitro inhibition of mushroom tyrosinase (Tyr)—(ΤyrI% (100 µM)); In vitro inhibition of trypsin induced proteolysis (IC_50_ (μM) or % Trypsin Inh—Iptr% (10 µM)).

Compd.	Clog *P* *	AAPH% 100 µM	LOX IC_50_ (μM) or % 100 μM	ΤyrI% 100 μM	IC_50_ (μM) or Iptr% 10 µM
**2a**	3.70	95	no	no	33
**2b**	6.25	79	no	no	8.25 μM
**2c**	3.34	91	no	16	10 μM
**2d**	4.86	81	49	5	7 μM
**2e**	2.87	60	15	3	60 μM
**2f**	5.80	no	no	nt	nt
**3a**	2.82	69	100 μM	nt	no
**3c**	2.47	93	39	7	6.75 μM
**3d**	3.99	91	37	nt	nt
**4a**	6.03	82	36	33	8 μM
**4b**	11.13	99	85 μM	12	9 μM
**4c**	5.33	93	41 μM	42	9.1 μM
**4d**	8.38	90	65 μM	28	8.5 μM
**4e**	4.39	83	88 μM	17	8.75 μM
**5c**	3.60	72	41	13	6.7 μM
**6c**	3.21	59	36	5	1 μM
NDGA		-	0.45 μM, 93	-	-
Trolox		93	-	-	-
Kojic acid		-	-	IC_50_ = 2.81 μM	-
Salicylic Acid					100 μM

* Theoretically calculated clog *P* values using the C-QSAR Program, Biobyte; no: no action under the experimental conditions; nt: not tested

**Table 2:** In vivo anti-inflammatory and analgesic activities of **4a** and **4c**. % inhibition of carrageenin-induced rat paw edema (CPE%) and % inhibition of writhing responses (writhing inhibition%).

Compd.	CPE (%) ^a^	Writhing Inhibition (%) ^a^
**4a**	44 *	32
**4c**	56 **	58
Indomethacin	58 **	-
Aspirin	-	77

* *p* < 0.01, ** *p* < 0.05; ^a^ Dose of the administered 0.0057 mmol/kg body weight.

**Table 3:** Molecular properties prediction-Lipinski “Rule of five. Drug likeness of the more potent representative compounds.

Compd.	milogP ^a^	TPSA ^b^	N^o^ Atoms	N^o^O,N ^c^	N^o^ OH, NH ^d^	N^o^ Violations	N^o^ Rotational Bonds ^e^	Volume ^f^	MW ^g^
**4a**	5.43	98.81	44	8	2	2	8	535.77	584.68
**4b**	9.17	117.28	63	10	2	2	15	782.76	954.72
**4c**	4.87	98.81	42	8	2	1	8	517.20	596.73
**4d**	7.47	98.81	53	8	2	2	9	640.59	684.80
**4e**	3.47	125.09	43	10	2	1	9	515.74	564.60
**5c**	3.57	49.41	24	4	1	0	4	309.90	340.45
**6c**	3.55	55.40	21	4	1	0	6	266.30	301.36

^a^ Logarithm of partition coefficient between-octanol and water (milogP); ^b^ Topological polar surface area (TPSA); ^c^ Number of hydrogen bond acceptors (n-ON); ^d^ Number of hydrogen bond donors (n-OHNH); ^e^ Number of rotatable bonds (n-rotb); ^f^ Molecular Volume; ^g^ Molecular Weight.

**Table 4:** Reaction data from the different synthetic methods of novel compounds.

Compd.	Method	Time (min)	Yield (%)	Compd.	Method	Time (min)	Yield (%)
**2a**	A	120	67	**3d**	E	15	60
**2a**	B	15	56	**4a**	F	75	12
**2b**	A	120	83	**4a**	G	10	94
**2c**	A	120	37	**4a**	H	4440	14
**2c**	B	15	66	**4b**	F	240	31
**2c**	C	20	19	**4c**	F	135	14
**2d**	A	120	53	**4c**	G	15	19
**2d**	B	15	38	**4c**	I	5	trace
**2e**	A	120	36	**4d**	F	90	10
**2f**	A	120	12	**4d**	G	20	92
**3a**	E	15	68	**4d**	I	5	trace
**3c**	D	120	26	**4e**	F	120	54

**Table 5:** Biological activities of the most potent derivatives.

Compd.	AAPH% 100 µM	LOX IC_50_ (μM) or % 100 μM	ΤyrI% 100 μM	IC_50_ (μM) or Iptr% 10 µM	CPE (%)	Writhing Inhibition (%)
**3c**	93	39	7	6.75 μM	-	-
**4a**	82	36	33	8 μM	44	32
**4b**	99	85 μM	12	9 μM	-	-
**4c**	93	41 μM	42	9.1 μM	56	58
**4d**	90	65 μM	28	8.5 μM	-	-
**4e**	83	88 μM	17	8.75 μM	-	-
**5c**	72	41	13	6.7 μM	-	-
**6c**	59	36	5	1 μM	-	-

## Abbreviations

AA: Arachidonic acid; AAPH: 2,2′-azinobis(2-amidinopropane) hydrochloride; BBB: Blood Brain Barrier; Clog *P*: Theoretical calculated lipophilicity; COX: Cycloxygenase; CPE: Carrageenin-induced rat paw edema; H(P)ETEs: 15-hydro(-pero)xy-eicosa-tetra-enoicacids; LOX: Lipoxygenase; LXs: Lipoxins; NDGA: Nordihydroguaretic acid; NSAIDs: non-steroidal anti-inflammatory drugs; PGs: Prostaglandins; QSAR: Quantitative Structure Activity Relationships; ROS: Reactive Oxygen Species; TEA: trimethylamine; W: Watt.
